# Autophagy plays a double-edged sword role in liver diseases

**DOI:** 10.1007/s13105-021-00844-7

**Published:** 2021-10-18

**Authors:** Jing-chao Zhou, Jing-lin Wang, Hao-zhen Ren, Xiao-lei Shi

**Affiliations:** 1 Nanjing Drum Tower Hospital, the Affiliated Hospital of Nanjing University Medical School, Nanjing, China; 2 Nanjing Drum Tower Hospital Clinical College of Traditional Chinese and Western Medicine, Nanjing University of Chinese Medicine, Nanjing, China

**Keywords:** Autophagy,·Liver diseases, Pathophysiology, Treatment

## Abstract

As a highly evolutionarily conserved process, autophagy can be found in all types of eukaryotic cells. Such a constitutive process maintains cellular homeostasis in a wide variety of cell types through the encapsulation of damaged proteins or organelles into double-membrane vesicles. Autophagy not only simply eliminates materials but also serves as a dynamic recycling system that produces new building blocks and energy for cellular renovation and homeostasis. Previous studies have primarily recognized the role of autophagy in the degradation of dysfunctional proteins and unwanted organelles. However, there are findings of autophagy in physiological and pathological processes. In hepatocytes, autophagy is not only essential for homeostatic functions but also implicated in some diseases, such as viral hepatitis, alcoholic hepatitis, and hepatic failure. In the present review, we summarized the molecular mechanisms of autophagy and its role in several liver diseases and put forward several new strategies for the treatment of liver disease.

## Introduction

The term “autophagy” is first identified by Christian René de Duve in 1963, and he is the first one who introduces the terms “endocytosis” and “phagocytosis” to designate pathways bringing components for digestion in lysosomes(48). Autophagy is a critical process for normal physiological events, which allows the lysosomal turnover of cellular energy metabolites, including degradation of intracellular organelles and specific proteins. As a dynamic recycling process, autophagy plays a vital role in the incessant renovation and homeostasis of cells(38).

There are three types of autophagy: macroautophagy, microautophagy, and chaperone-mediated autophagy, which are named according to their different mechanisms. Macroautophagy is the most usual class that relies on the “autophagosome,” a spherical vesicle in the cell with the ability to move to the microtubule-organizing center (47). Subsequently, the autophagosome becomes an autolysosome, which can degrade the materials contained within it after fusing with the lysosome. In the second type of autophagy, microautophagy, the lysosome encapsulates small intracellular substrates by inward invagination of the lysosomal membrane. As for the last type, chaperone-mediated autophagy, substrates are transported across the lysosomal membrane directly. There are chaperones and cochaperones in the membrane, such as heat shock cognate 70 (HSC70). The specific recognition in this process depends on cytosolic proteins containing a lysine-phenylalanine-glutamate-arginine-glutamine (KFERQ)-like pentapeptide (45). The transmembrane protein Lamp-2A and unfolded proteins are transported into the lysosome through a multimeric translocation complex.

**Fig. 1:**
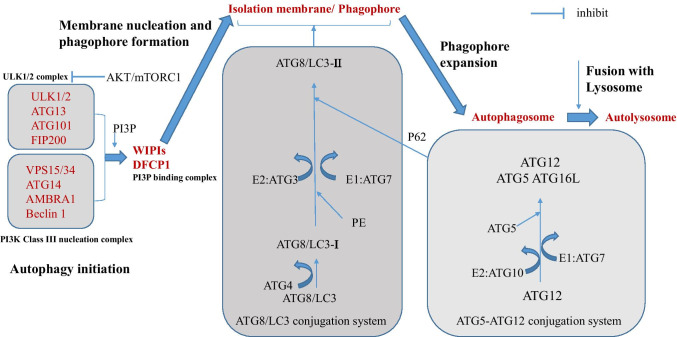
Process of Autophagy. There are three steps in the formation stage of the autolysosome: (1) autophagy initiation, (2) membrane nucleation and phagophore formation, (3) phagophore expansion and autophagosome formation, and (4) fusion with lysosome and autolysosome formation

It is considered that macroautophagy is the main class of autophagy, and there are more in-depth investigations about this type compared with the others. Therefore, macroautophagy is simply regarded as ‘‘autophagy.’’ Similarly, programmed cell death (PCD) pathways, such as apoptosis and regulated necrosis, also play a vital role in normal cell renewing and homeostasis of the tissue. Apoptosis is also one of the normal processes, in which some physiological factors induce PCD. Compared with necrosis in which cell death occurs usually because of some serious physicochemical extracellular factors accompanied by inflammatory responses, the apoptotic process begins without any inflammatory response and generally starts from the inside of the cell, and this is an active process accompanied by the energy consumption. Moreover, some specific proteins would be synthesized during the apoptotic process. Autophagy is greatly induced during starvation or under other highly stressful conditions, leading to a rapidly increased number of autophagosomes. This pathway involves more than 30 autophagy-related genes (ATGs), which is relatively complex (Fig. 1). The functions of Atg8/LC3, Atg7, and Atg6/Beclin 1 are among the best characterized in mammalian cells. Atg6/Beclin 1 forms a complex with the class III phosphoinositide 3-kinase (PI3K) complex, VPS34, and VPS15, leading to autophagy-specific generation of phosphatidylinositol-3-phosphate (PtdIns3P). PtdIns3P is required for a series of events that ultimately contribute to the conjugation of Atg8/LC3 to phosphatidylethanolamine (PE) at the autophagosomal membranes, which also requires Atg7. PE-conjugated Atg8/LC3, also known as LC3-II (in contrast to the unlipidated LC3-I), is important in autophagosome formation(37). Several recent studies have suggested that additional membranes that contribute to the formation of autophagosome are also originated from the Golgi complex, the mitochondria, and the plasma membrane beside the endoplasmic reticulum (ER)(39). ATGs play a vital role in this process. Previous research in yeast has demonstrated that not all the 35 ATG proteins are the ‘‘core ATG proteins’’ and only Atg1–10, 12–14, 16, and 18 play a vital role in the formation of autophagosome(41).

## The physiological function of autophagy in liver

As a katabolic process, autophagy plays a key role in the maintenance of hepatocellular and tissue homeostasis. Autophagy’s contribution to the production of amino acids is increased when cells are starved, showing that autophagy is a necessary survival response against starvation conditions [59]. Furthermore, autophagy plays a crucial role in lipid metabolism, namely lipophagy. In mice with specific depletion of ATG7 or administered with the autophagy inhibitor 3-methyladenine (3MA), the contents of liver triglyceride and cholesterol are significantly increased [60]. Meanwhile, mitophagy, the selective autophagy of mitochondria, is suggested to be a major degradation mechanism, by which mitochondria turn over remove the damaged mitochondria and eliminate mitochondrial cytoplasmic remodeling under nutrient-rich conditions [21]. Moreover, reactive oxygen species (ROS) can lead to mutations in mitochondrial DNA (mtDNA) that lacks histones, and mitochondria have limited DNA repair capacity compared with the nucleus, making mtDNA more vulnerable to oxidative damage. The damaged or mutated mtDNA would be degraded by mitophagy. Therefore, mitophagy is necessary for keeping hepatocellular homeostasis. Moreover, mitophagy has additional physiological functions, such as the regulation of apoptosis, circadian rhythm, and immune response [3].

## Role of autophagy in several hepatic diseases

### Liver cancer

Hepatocellular carcinoma (HCC) ranks sixth among the common types of human cancer worldwide in recent years [34]. Increasing evidence has shown that autophagy plays an essential role in different stages of liver cancer. Several studies have suggested that autophagy has anti-liver cancer effects. First, autophagy can stop hepatic cells from canceration by enhancing the anti-tumor effect of IFN-γ [25]. Furthermore, release of high-mobility group box 1 (HMGB1) induced by the inhibition of autophagy in hepatocytes leads to tumor development [20]. Similarly, a recent study has demonstrated that dihydroartemisinin (DHA), not only used as an autophagy accelerant but also a substance with anti-carcinogenesis effects, can suppress the proliferation of HCC cells in a dose- and time-dependent manner, and the restraining effect is mediated by autophagy [52]. These studies indicate that autophagy acts as a barrier that inhibits liver cancer occurrence.

**Fig. 2:**
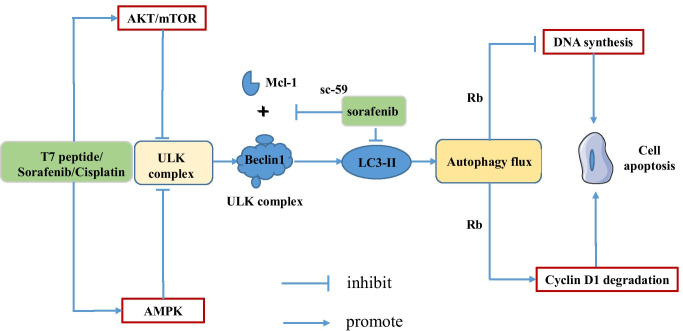
Autophagy plays an antitumor role in liver cancer. Sorafenib resistance and autophagy in HCC. Sorafenib inhibits autophagy by activating ATK/mTOR and AMPK pathway, resulting in drug resistance. Sorafenib inhibits autophagy process by changing multiple ATGs activity. In addition, sorafenib and its novel derivative, sc-59, inhibit the binding of Mcl-1 to Beclin 1. Autophagy is associated with apoptosis through protein Rb, and the formation of LC3-II is interfered by sorafenib, causing autophagy inhibition and drug resistance that inhibit cell apoptosis

Interestingly, it has been verified that autophagy is involved in cancer metastasis [17]. It plays a critical role during liver cancer progression by promoting hepatocarcinoma cells survival and proliferation, in addition to other oncogenic effects such as induction of EMT and promotion of cell migration, invasion, and metastasis. For example, LncRNA MCM3AP-AS1 directly interacts with miR-455, in turn regulating autophagy to promote hepatocellular carcinoma metastasis [66]. Autophagy also can lead to drug resistance in HCC which may cause therapeutic failure. For instance, sorafenib has a good anti-cancer effect, but many middle and late stage patients cannot escape the fate of drug resistance. A recent research has demonstrated that hypoxia significantly attenuated sensitivity of HCC cells to sorafenib treatment [28]. Higher level of autophagy was observed in sorafenib-treated hepatoma cells under hypoxia condition, and inhibiting autophagy eliminated hypoxia-induced drug resistance. Their finding suggested that hypoxia-induced autophagy is the main mechanism mediating sorafenib resistance in HCC cells and FOXO3a plays a key role in regulating hypoxia-induced autophagy [28]. Furthermore, Chu et al. have found that folate receptor α (FOLR1) was significantly upregulated in drug-resistant HCC cells and their further research demonstrated that FOLR1-induced autophagy may be a main mechanism mediating sorafenib resistance in HCC cells [9]. Similarly, the T7 peptide plays a growth inhibition role in multiple types of carcinoma cells and Liu et al. have introduced that autophagy may have a protective function in the process of T7 peptide-induced apoptosis in human HCC cells [30], which indicates that targeting autophagy to sensitize cancers may be an effective therapeutic strategy to overcome drug resistance. Furthermore, the mitochondrial-lysosomal crosstalk is constituted with cisplatin-induced mitophagy and lysosomal biogenesis, and it is a critical mechanism, by which HCC cells develop drug resistance to cisplatin [51]. Figure 2 describes the main mechanism that autophagy leads to drug resistance.

These findings demonstrate that the same autophagy-related pathway can choose self-existence dynamically and changefully and have a bidirectional function. In general, autophagy has tumor suppressor functions in normal hepatocytes by maintaining the normal function of liver cells. After the tumor formation, it promotes the survival of HCC cells or makes them develop more malignant behaviors in the tumor microenvironment. So further researches may focus on how to optimize the combination of autophagy inhibition and anti-tumor drugs.

### Liver fibrosis

**Fig. 3:**
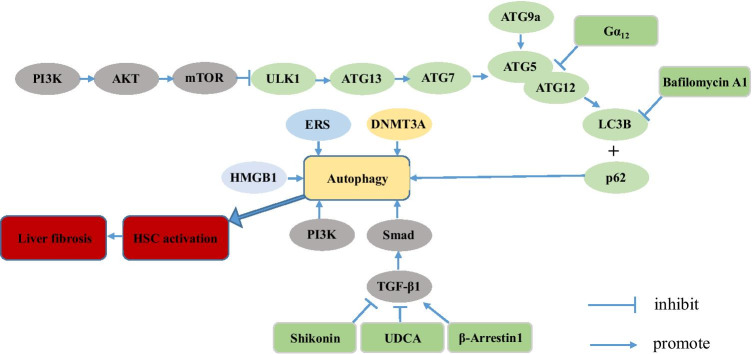
Autophagy plays a pro-fibrosis role. Autophagy promotes liver fibrosis through multiple pathways: (1) The TGF-β1/Smad signaling pathway; (2) the NF-κB signaling pathway; (3) the PI3K/AKT/mTOR signaling pathway; and (4) the Hedgehog signaling pathway

Hepatic stellate cells (HSCs) play a crucial role in the pathogenesis of hepatic fibrosis, which is protective and reversible [16]. Activated HSCs undergoing morphological and functional changes are transformed into fibroblasts or myofibroblasts, expressing plenty of abundant α-smooth muscle actins [19]. Several pieces of research have demonstrated that autophagy also plays an essential role in such a process through multiple pathways (Fig. 3). Many studies have demonstrated that autophagy promotes digestion of lipid droplets in quiescent HSC, thereby facilitating HSC activation [14, 58]. Follow-up mechanistic studies demonstrated that endoplasmic reticulum stress via the XBP1 arm of the unfolded protein response, oxidative stress or G proteins (G aplha 12) promote HSC activation by enhancing autophagy, resulting in enhanced fibrosis [15, 22, 23]. In addition to lipophagy, Tan et al. have introduced that β-arr1, a scaffolding protein that regulates G protein-coupled receptor (GPCR) signaling transduction, promotes liver fibrosis via autophagy-mediated Snail signaling [56]. Furthermore, several studies have been carried out to identify whether inhibition of autophagy can be a new therapeutic strategy to prevent liver fibrosis. Ye et al. have demonstrated that inhibition of autophagy contributes to anti-fibrotic effect of ursodeoxycholic acid (UDCA) in rats with liver fibrosis [64]. Shikonin can attenuate CCl4-induced liver fibrosis in mice by restraining plasminogen activator inhibitor-1[13]. Further research has demonstrated that shikonin attenuates liver fibrosis by downregulating the transforming growth factor-β1/Smads pathway and inhibiting autophagy [31]. Taken together, further exploration is necessary and downregulating the level of autophagy may become a new strategy for the treatment of liver fibrosis.

Although a number of researches have shown that autophagy promotes the development of hepatic fibrosis, some studies have indicated that it could also inhibit hepatic fibrosis. For example, Ni et al. found that loss of autophagy in hepatocytes causes cell death resulting in liver fibrosis, because of proteotoxicity and disruption of pro- and anti-apoptotic protein homeostasis [43]. Furthermore, autophagy is an antiphlogistic pathway in macrophages from multiple source, including Kupffer cells, by inhibiting production of IL1-β, a survval and profibrogenic pathway for HSC. Co-culture of ATG5-deficient Kupffer cells with HSC increase the fibrogenic properties of HSC, and this effect was attenuated in the presence of IL1-R antibody [32]. Similarly, several studies have been carried out to identify whether activation of autophagy can be a new therapeutic strategy to prevent liver fibrosis. Oroxylin A shows multiple pharmacological effects, such as antiphlogistic and tumor-suppressor functions [26, 44]. Chen et al. have explored the effect of oroxylin A on liver fibrosis, and their study shows that oroxylin A attenuates CCl4-induced liver fibrosis and decreases the number of alleviated HSCs by modulating autophagy pathway [6].

To sum up, autophagy is undoubtedly the main participant in liver fibrosis. However, because autophagy promotes HSC activation but generates anti-fibrogenic signals via its hepatoprotective and anti-inflammatory effects [1] in hepatocytes, macrophages and endothelial cells, targeting autophagy for anti-fibrogenic therapies will require cell type specific approaches.

### Liver injury

Liver injury is characterized by inflammation-mediated liver cell damage, accompanied by apoptosis and necrosis. Liver injury can be secondary to multiple liver diseases, such as hepatitis, ischemia–reperfusion, improper use of drugs, and so on. Patients with acute liver injury may progress to acute liver failure (ALF) which is clinically characterized by rapid worsening of hepatic function, resulting in high mortality and morbidity [54]. Several studies suggest that protection of autophagy is crucial in liver injury caused by multiple challenges, including tumor necrosis factor-α (TNFα), acetaminophen (APAP), hepatic ischemia–reperfusion injury (IRI), and an overabundance of fatty acids [4, 18, 55]. There have been several studies indicating that the sheltering effect on APAP-induced liver injury is indivisible from autophagy. Recombinant IL-22 was reported to have a protective effect in APAP-induced acute liver injury [50]. Subsequently, Mo et al. demonstrated that autophagy in AMPK-dependent manner was involved in the hepato-protective effect of IL-22 against hepatic injury induced by APAP [40]. Similarly, Ni et al. demonstrated that the induction of autophagy by rapamycin can prevent, while the inhibition of autophagy by chloroquine can exacerbate, APAP-induced liver injury in mice [42]. These findings indicate that a high levels of autophagy can attenuate the toxicity of APAP. In addition, a number of plant components also are involved in in repairing liver damage by regulating autophagy. Dihydroquercetin repaired all the damages induced by APAP by activating autophagy and phosphorylation of the Janus kinase 2/signal transducer and activator of transcription 3 (JAK2/STAT3) cascade, which subsequently inhibited ROS accumulation, mitochondrial dysfunction, extracellular signal-regulated kinase-JNK stress and necrosis [65]. Glycycoumarin, which is purified from licorice, is able to attenuate APAP-induced oxidative stress and liver injury by activating protective autophagy and the JNK signaling pathway [61]. These findings may represent a novel approach for the treatment of drug-induced hepatotoxicity by pharmacologically elevating or restoring autophagy function to improve the liver function.

### Viral hepatitis

Chronic HBV or HCV infection may cause serious liver diseases, such as hepatocellular cancer, and cirrhosis [2, 35]. HBV replication is regulated via multiple intracellular or extracellular factors, including specific hormones, inflammatory factors, and intracellular specific pathways [27, 49, 63]. Autophagy as an innate cellular immunity plays a crucial role in pathogen clearance [10]. A previous study has proved that HBV replication is also regulated via autophagy [7]. Chen et al. have explored the function of miR-155 in HBV replication and demonstrated that miR-155 enhances HBV replication by reinforcing the SOCS1/Akt/mTOR axis-stimulated autophagy [8]. As for the replication of HCV, a recent study has shown that interaction between non-structural protein 5A and ATG14L as well as the ATG14L-Beclin1-Vps34Vps15 complex is crucial for the formation of autophagosomes. Furthermore, it suggests that ATG14L is necessary for the creation of HCV double-membrane vesicles (DMVs) and viral RNA replication [24]. In addition, many researchers demonstrated that alcohol has a significant promoting effect on HCV-related liver diseases. Subsequently, Ran et al. have demonstrated that alcohol promotes HCV replication by up-regulation of several important autophagic factors, including ATG7 and ATG5–ATG1 [46]. These studies may provide some clues for new treatments to improve the curative effect of antiviral agents among patients with viral hepatitis.

### Steatosis and fatty liver diseases

The health issue induced by excess intake of fat becomes more and more severe, which is the main cause of metabolic associated fatty liver disease or “MAFLD”(formerly known as NAFLD) [36, 67]. Disrupted hepatic lipid homeostasis resulting in hepatic triglyceride accumulation is a hallmark of NAFLD. Therefore, controlling general hepatic lipid accumulation is essential to prevent or reverse progression of NAFLD. For example, ATG knockdown or pharmacological inhibition of autophagy accelerates lipid accumulation [53]. Similarly, overexpressing ATG7 suppresses development of hepatic steatosis in high-fat-diet-fed mice [57]. Several studies suggested that autophagy plays an anti-steatogenic role in liver cells via lipophagy. Lipophagy controls the breakdown of lipid droplets(LDs), a form of autophagy specifically involved in the degradation of LDs, which has been identified as a new pathway that also contributes to NAFLD [12, 33]. A recent study indicated that advanced stage NAFLD is associated with greater damages of hepatic autophagy [5] and there is also an significantly association between the autophagy-related GTPase family M (IRGM) gene and increased susceptibility of NAFLD in obese children [29]. In vitro experiments indicated that IRGM knockdown downregulated the autophagic flux and increased LD content, while overexpression of IRGM downregulated LD content, highlighting its function in lipophagy. Accordingly, blockade of autophagy by pharmacological inhibitor or downregulating expression ATGs caused the retention of triglycerides and lipid droplets [53], reduced free fatty acid oxidation, and lowered the secretion of very-low-density lipoprotein (VLDL) from liver cells. In addition, a recent study has demonstrated that ghrelin isoforms have protective effects on NAFLD, and the functions are mediated by their capacity to reduce TNF-α-induced liver cell apoptosis, pyroptosis, and autophagy [11]. Moreover, the elimination levels of liver fat are positively correlated with autophagy, while downregulation of autophagy can lead to accumulation of lipids [62]. Further study suggests that protein kinase C-ε (PKC-ε) may participate in liver cell autophagy through controlling PI3K [62]. These experimental results suggest a new treatment of fatty liver disease. However, the complexity in the cellular composition of the liver and the diversity in the response of these cells to pathological or physiological stimuli may need a cell type-specific strategy for modulating autophagy function to achieve the beneficial effect.

## Conclusions

**Fig. 4:**
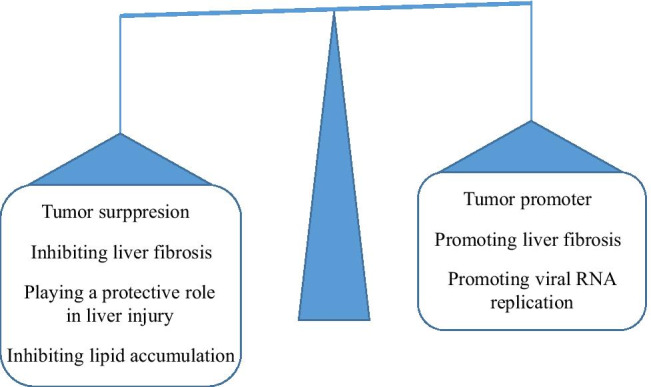
Autophagy in liver diseases the two faces of Janus

To maintain cellular homeostasis and normal functions of tissues, cells routinely renew their components through some specific processes, such as autophagy. In this review, we summarized various physiological and pathological functions of autophagy in the liver. As shown in Table 1 and Fig.4, autophagy has bidirectional functions in hepatic diseases, making it attractive but challenging to apply autophagy to the treatment of liver diseases in clinical practice. There is no way to determine the efficiency of autophagy because no biomarker is available for now. Furthermore, a more specific drug that can modulate the activity and type of autophagy is needed if we want to specifically target autophagy in the liver, which has become a strategy for the treatment of hepatic diseases in the future. Therefore, it is necessary to solve the above-mentioned problems in further investigations.

**Table 1:** Autophagy in liver diseases the two faces of Janus

**Liver diseases**	**The autophagic effect in liver diseases**	
Liver cancer	Inhibiting proliferation of hepatocellular carcinoma [13–15]	Accelerating drug resistance of T7 peptide, sorafenib and cisplatin [1–21] Reverting miR455-inhibited metastasis of HCC cells [17]
Liver fibrosis	Loss of autophagy in hepatocytes causes cell death resulting in liver fibrosis [35] Inhibiting liver fibrosis by changing the survival and profibrogenic pathway for HSC [36]	Promoting digestion of lipid droplets in quiescent HSC, thereby facilitating HSC activation [26–28] Promoting liver fibrosis via autophagy-mediated Snail signaling [29] Inhibiting autophagy contributed to the anti-fibrotic effect [30]
Liver injury	Playing a protective role in liver injury caused by multiple challenges induced by APAP, IRI [31–46]	-
Viral hepatitis	-	Strengthening autophagy can promote viral RNA replication [53–56]
Steatosis and fatty liver diseases	Reducing the accumulation of liver fat [59–66]	-

## Conflict of interest

The authors declare no conflicts of interest.

## Abbreviations

ADH: Alcohol dehydrogenase
ALD: Alcoholic liver disease
ALF: Acute liver failure
APAP: Acetaminophen
ATGs: Autophagy related genes
DHA: Dihydroartemisinin
DMV: Double-membrane vesicles
EMT: Epithelial-Mesenchymal Transition
ER: Endoplasmic reticulum
FOLR1: Folate receptor α
GPCR G: Protein-coupled receptor
HBV: Hepatitis B virus
HCC: Hepatocellular carcinoma
HCV: Hepatitis C virus
HMGB1: High-mobility group box 1
HSC70: Heat shock cognate 70
HSCs: Hepatic stellate cells
IRI: Ischemia–reperfusion injury
LDs: Lipid droplets
mtDNA: Mitochondrial DNA
NAFLD: Nonalcoholic fatty liver disease
PCD: Programmed cell death
PKC-ε: Protein kinase C-ε
PTE: Pterostilbene
ROS: Reactive oxygen species
TNFα: Tumor necrosis factor-α
UDCA: Ursodeoxycholic acid
VLDL: Very-low-density lipoprotein
